# Ethnopharmacological Approaches to Wound Repair

**DOI:** 10.1155/2013/804039

**Published:** 2013-02-27

**Authors:** Esra Küpeli Akkol, Fatma U. Afifi, Saringat Hj. Baie, Ashok D. Taranalli, Shrikant Anant

**Affiliations:** ^1^Department of Pharmacognosy, Faculty of Pharmacy, Gazi University, 06330 Ankara, Turkey; ^2^Faculty of Pharmacy, University of Jordan, Amman 11942, Jordan; ^3^Department of Pharmaceutical Technology, School of Pharmacy, Universiti Sains Malaysia, 11800 Minden, Malaysia; ^4^Department of Pharmacology, Faculty of Pharmacy, KLE University, Belgaum, Karnataka 590010, India; ^5^Department of Molecular and Integrative Physiology, and Medicine, University of Kansas School of Medicine, 3901 Rainbow Boulevard, MS 3040, Kansas City, KS 66160, USA

Wound is breaking of the skin by a physical injury. Wound healing is a connective tissue response along with the repair process which immediately comes after the injury. It occurs as a sequence of phases such as haemostasis, inflammation, proliferation, and remodelling and causes series of interactions between the extracellular matrix, cytokine mediators, and different cell types. For rapid healing several medicinal plants were reported in ethnobotanical studies. Traditional remedies which claimed to have wound healing potential are widely used in developing countries due to their accessibility and low cost. However, these remedies should be evaluated for their efficacy and safety before their utilization. In this context, the papers selected for this special issue include scientifically evaluated information and lead to development of novel drugs for rapid healing of wounds. We would like to thank the authors for their contributions for this special issue.

 This special issue contains twelve papers. T. Lin et al. investigated the wound healing effect of tocopherol in diabetic rats. This study has proven the wound healing potential of tocopherol cream by increasing the rate of wound closure and total protein content significantly in diabetic condition.

 R. Samy and V. Chow provide a scientific basis for the use of *Calotropis procera *for treating skin and wound infections in traditional medicine. The aqueous extract of stem bark of *C. procera *exhibited more pronounced potent antimicrobial activity. Calo protein isolated from the aqueous extract of *C. procera *showed broad-spectrumactivity as well as significant wound healing activity.

 In the paper entitled “*Plectranthus amboinicus and Centella asiatica cream for the treatment of diabetic foot ulcers*,” Y. Kuo et al. investigated the effects of a topical cream containing *P. amboinicus *(Lour.) Spreng. (Lamiaceae) and *C. asiatica *(L.) Urban for diabetic foot ulcers.* P. amboinicus *and *C. asiatica *cream was found to be a safe alternative to hydrocolloid fiber dressing without significant difference in effectiveness.

 F. Li et al. used an *in vitro* model of ulcer-like wound processes, lithium-chloride-(LiCl-) induced cultured mouse keratinocytes, to investigate the effects of astragaloside IV treatment, and they concluded that astragaloside IV can promote ulcerated wound healing by downregulating *β*-catenin to increase keratinocyte migration and proliferation.

F. Li et al. discuss the classification and pathogenic process of chronic skin ulcers and strategies of traditional Chinese medicine. This study has shown a good approach to wound management by means of the strategies of traditional Chinese medicine for different wound types.

 The results of the paper by S. Yu and L. Yu entitled “*Dexamethasone resisted podocyte injury via stabilizing TRPC6 expression and distribution*” revealed that dexamethasone may maintain the structure and function integrity of slit diaphragm by blocking TRPC6 signal pathway and played an important role in mechanisms of antiproteinuria.

 C. Y. Hisao et al. investigated the wound healing effect of *Angelica sinensis *in the paper entitled “*A study of the wound healing mechanism of a traditional Chinese medicine, Angelica sinensis, using a proteomic approach*.” The wound healing potential of *Angelica sinensis* was confirmed by proteomic and biochemical analysis in scientific platform.

 M. Seelinger et al. showed the antineoplastic and wound healing potential of *Pluchea odorata *according to the bioactivity-guided fractionation assay.

 The other paper was on the inhibitory activity of *Nelumbo nucifera* (Gaertn.) on the development of atopic dermatitis by Karki et al. The results of the study suggested that *Nelumbo nucifera* (Gaertn.) leaf may be a useful natural resource for the management of atopic dermatitis, which is a chronic inflammatory skin disease.

 S. Park et al. evaluated the healing effect of *Chrysanthemum indicum* L. on skin lesions. This study revealed that *Chrysanthemum indicum* reduced interleukin- (IL-) 4 and IL-13 in 2,4-dinitrochlorobenzene-treated HaCaT cells and may be an effective alternative substance for the management of the atopic dermatitis.

 The paper, by L. Parente et al., evaluated the wound healing and anti-inflammatory activity of *Calendula officinalis* in animal models. This experimental study revealed that *C. officinalis* possesses anti-inflammatory and antibacterial activities as well as angiogenic and fibroblastic properties acting in a positive way on the inflammatory and proliferative phases of the healing process.

 And the paper by I. Tumen et al. evaluated the wound healing and anti-inflammatory activities of the essential oils obtained from some *Juniperus* species, growing in Turkey, by using linear incision and circular excision experimental wound models, hydroxyproline estimation, and acetic-acid-induced capillary permeability tests. The results showed that *J. oxycedrus *subsp. *oxycedrus* and *J. phoenicea* display remarkable wound healing and anti-inflammatory effects which support the folkloric use of the plants.

*Esra Küpeli Akkol* *Esra Küpeli Akkol* *Fatma U. Afifi* *Fatma U. Afifi* *Saringat Hj. Baie* *Saringat Hj. Baie* *Ashok D. Taranalli* *Ashok D. Taranalli* *Shrikant Anant* *Shrikant Anant*

